# Targeting p38α in cancer: challenges, opportunities, and emerging strategies

**DOI:** 10.1002/1878-0261.70204

**Published:** 2026-01-12

**Authors:** Angel R. Nebreda

**Affiliations:** ^1^ Institute for Research in Biomedicine (IRB Barcelona) The Barcelona Institute of Science and Technology Spain; ^2^ ICREA Pg. Lluís Companys 23 Barcelona Spain

**Keywords:** cancer cell viability, cancer therapy, context‐dependent signaling, p38α (MAPK14), tumor microenvironment

## Abstract

The protein kinase p38α is an important regulator of cell homeostasis that has been implicated in the response to many types of stresses. Given the plethora of functions that can be potentially regulated by p38α, this pathway has been linked to many diseases including cancer, suggesting a potential therapeutic interest in targeting p38α. This Viewpoint focuses on the role of p38α stress signaling in cancer development and therapy, discussing recent reports and reflecting on future challenges.

## Graphical abstract

p38α normally regulates cellular stress responses and homeostasis and suppresses malignant transformation. In cancer, however, p38α is co‐opted to drive context‐dependent proliferation and dissemination. p38α also supports key functions in cells of the tumor microenvironment, including fibroblasts, myeloid cells, and T lymphocytes. Together, p38α activity in cancer and stromal cells promotes tumor progression and therapy resistance, suggesting that targeting this pathway may enhance anticancer therapies. Image created with BioRender.com.

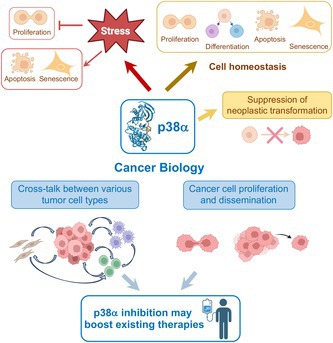

**Abbreviations**
EGFR: Epidermal growth factor receptor
KO: knockout
MAPK: mitogen‐activated protein kinase
SASP: senescence‐associated secretory phenotype

## 1 Introduction

Cell survival requires the ability to deal with different types of stressful situations in a precise and coordinated manner. Various signaling networks have evolved to allow cells to sense and adapt to environmental changes. These signals often involve reversible post‐translational modifications, which may alter protein activity, stability, or interactions, ultimately shaping cellular behavior.

The mitogen‐activated protein kinases (MAPKs) are among the most evolutionary conserved signaling modules. Within this family, p38α (encoded by the *MAPK14* gene) is a ubiquitously expressed protein kinase with essential functions in cell homeostasis. Genetic studies in mice have shown that p38α is critical for placental organogenesis, with p38α knockout (KO) mice exhibiting embryonic lethality or perinatal death due to lung developmental defects [1]. Functionally, p38α can regulate a wide spectrum of cellular processes by phosphorylating multiple substrates, including transcription factors and chromatin regulators that can either activate or inhibit gene expression, as well as different types of cytosolic proteins involved in apoptosis, cytoskeletal remodeling, protein degradation, mRNA stability, or cell migration [1, 2, 3, 4].

Because of this versatility, p38α serves as a central hub in coordinating cellular responses to physiological and pathological stimuli. It can integrate signals from environmental stresses, inflammatory cues, and intracellular dysfunctions to control cell cycle progression, differentiation, apoptosis, senescence, and immune responses. Importantly, this broad regulatory capacity also links p38α signaling to several human diseases including cancer, where it can exert context‐dependent functions. This article examines the diverse roles of p38α in cancer biology, highlights recent exciting findings, and discusses the challenges that must be addressed to fully realize the therapeutic potential of targeting p38α.

## 2 Cancer cell regulation

Initial studies using cancer cell lines and genetic mouse models of several cancer types provided evidence that p38α signaling can suppress tumor initiation, typically by interfering with malignant transformation of the epithelial cells. However, strong evidence also indicates that p38α contributes to cancer cell functions that sustain primary tumor growth in mouse models and promote the dissemination of certain cancer cell types. The pro‐tumorigenic roles of p38α are mediated by different mechanisms, which seem to be context‐dependent [5, 6, 7]. It is still unclear what makes certain types of cancer cells more dependent on p38α functions to maintain their survival, proliferation, or spreading properties. This dependence does not seem to be connected with the tissue of origin of the tumor, and a possible link with particular genetic make‐ups of the cancer cells has also remained elusive. It should be noted that p38α activation by itself is unlikely to induce neoplastic transformation. Instead, p38α appears to help maintain homeostasis by enabling cells to adapt to the metabolic and regulatory changes that support cancer cell survival and expansion. This probably involves a switch in the p38α‐regulated functions from nonmalignant cells to established cancer cells. For example, it has recently been shown that a new type of p38α inhibitor named ULTR‐p38i induces uncontrolled mitotic entry in cancer cells, which leads to mitotic catastrophe resulting in apoptosis or senescence [8].

A recent report has illustrated a role for p38α in the growth and tumorigenicity of high‐grade endometrial carcinoma spheroids, while having no effect on conventional 2D monolayer cultures [9]. Evidence is presented that p38α probably contributes to cancer stemness characteristics. Previous work has also implicated p38α in the proliferation of lung cancer cells in 3D models but not in 2D cultures [10]. In both cases, mouse experiments support the hypothesis that p38α in cancer cells promotes tumorigenicity *in vivo*, supporting the relevance of 3D models to study malignant pathology. It appears that cancer cells grown in 3D models activate biological programs that are more dependent on p38α signaling than those activated in monolayer cultures, providing an interesting perspective to the context dependent role of p38α, which could be linked to cancer stemness markers.

The p38α functions can be tuned by several types of post‐translational modifications beyond the activation loop phosphorylation that is critical for its kinase activity [5]. A new mechanism has been proposed that regulates p38α phosphorylation in lung cancer cells and involves methylation at Lys165 [11]. Interestingly, the demethylase KDM5D can target this lysine, whose demethylation reduces p38α phosphorylation and represses cancer cell proliferation in various cell lines and xenograft models of lung cancer. This work illustrates how the presence of specific post‐translational modifications that impinge on p38α activity, together with the expression of enzymes that can regulate these post‐translational modifications, may influence the contribution of p38α to tumor progression [11].

Another important consideration is the ability of p38α to regulate a pathway or process at several levels, sometimes exerting opposing effects. Consequently, the outcome of p38α activation likely depends on the presence of other cellular signals that converge on the same targets or processes. For instance, while p38α may influence β‐catenin stability and activity through different mechanisms, its overall impact on Wnt/β‐catenin signaling will ultimately depend on the status of other intersecting pathways [12, 13].

All in all, the extensive cross‐talk of p38α with other signaling pathways together with the potential concurrent regulation of different cellular processes by p38α gives a lot of versatility to this pathway, but it also makes it difficult to anticipate the effect expected upon tampering with p38α activity.

## 3 Tumor microenvironment

In addition to the cancer cells, the tumors also contain a number of nonmalignant cell types. There is evidence implicating p38α signaling in the communication between different cells of the tumor, by acting either in the cancer cells or in fibroblasts and immune cells. Cancer cells rely on p38α to produce chemokines and cytokines that facilitate the recruitment of protumoral myeloid cells to the tumor niche and decrease tumor infiltration by cytotoxic CD8+ T cells. This has been reported in breast cancer models in which p38α inhibition reduces tumor growth and lung metastasis [14]. More generally, p38α is an inducer of the senescence‐associated secretory phenotype (SASP), which can promote tumorigenesis at different levels [15]. In addition, p38α has been implicated in the upregulation of the T‐cell inhibitory protein PD‐L1 [16] and the immunosuppressive protein CD73 [17] by cancer cells, which dampen T‐cell activity favoring tumor immune evasion. Taken together, these results indicate that, in addition to its cancer cell‐specific functions, p38α also helps to shape the tumor microenvironment and restricts antitumor immunity.

Nonmalignant cells of the tumor stroma rely on p38α signaling to perform important functions. These include several protumorigenic roles of fibroblasts such as triggering the production of protumorigenic SASP factors [18], remodeling the extracellular matrix [19], and inducing the expression of cytokines and chemokines that mobilize glycogen in cancer cells [20] or that enable lung infiltration by neutrophils [21]. The activation of p38α in fibroblasts is usually induced by cancer cell‐secreted extracellular factors.

The p38α pathway has been shown to promote protumorigenic functions in immune cells. Accordingly, genetic downregulation of p38α in macrophages or dendritic cells reduces inflammation‐driven colon tumorigenesis in mouse models [22, 23]. In addition, p38α inhibition boosts antitumor immune responses by augmenting the T‐cell stimulatory capacity of dendritic cells [24]. There is also evidence that genetic or pharmacologic blockade of p38α in T cells can enhance antitumor immune responses, either by stabilizing IFNAR1, improving the survival of cytotoxic T cells [25] or by enhancing the potency of adoptively transferred T cells, endowing them with more durable and polyfunctional responses [26].

Insights into how p38α in different cell populations controls tumor growth is often obtained by using mice with p38α specifically deleted in particular cell types or by using cocultures of cancer cells with other cell types isolated from tumors. However, systemic administration of p38α inhibitors is likely to block p38α activity in most cells in the tumor. Accordingly, p38α inhibitors have been shown to limit tumor growth by reprogramming the metastatic tumor microenvironment in a CD4^+^ T cell‐, IFNγ‐, and macrophage‐dependent manner. It remains unclear which specific tumor cell populations require p38α inhibition to achieve this effect, but it could be the result of coordinated inhibition across several cell types [27]. Altogether, the information available supports the idea that p38α signaling in different cell types of the tumor ecosystem frequently facilitates tumor development.

## 4 Chemotherapy, targeted therapy and immunotherapy

The p38α pathway has been linked to the response to chemotherapeutic drugs in cancer cell lines by controlling several mechanisms that can either promote or antagonize cytotoxicity depending on the drug [6, 28, 29]. In some cases, p38α inhibition has been reported to produce diverse effects when the same drug is tested across different cancer cell lines. In general, the ability of p38α to regulate cell cycle checkpoints and DNA repair at different levels is likely to contribute to the sensitization of cancer cells treated with p38α inhibitors to drugs that directly or indirectly induce DNA damage, such as cisplatin, etoposide, or taxanes. Considering the functions of p38α in the tumor stroma, it is expected that *in vivo* models should be better suited to predict the effect of p38α inhibition on the response to cancer therapies. In this line, the use of chemical inhibitors and mouse genetic models has shown that targeting p38α potentiates the antitumoral effect of both cisplatin and taxanes in mouse models of breast cancer [30, 31].

Beyond chemotherapy, p38α signaling has been implicated in the cellular response to ionizing radiation, with potential implications for radiotherapy treatments in the clinic [32, 33]. In addition, there are convincing reports showing the interest of combining inhibitors of p38α with clinically used targeted anticancer therapies, such as the multikinase inhibitor sorafenib in hepatocellular carcinomas [34], Smac mimetics in leukemia [35], and checkpoint kinase 1 inhibitors in KRAS or BRAF mutant tumors [36]. Recent work has also provided evidence that melanoma cells treated with a BRAF inhibitor activate p38α, which induces the expression of the drug efflux transporter ABCG2, facilitating melanoma cell survival and the acquisition of resistance to BRAF inhibition [37]. Similarly, p38α inhibition has been shown to chemosensitize BRCA1‐deficient mammary cancer cells that have developed resistance to PARP inhibitors. This effect is mediated by a newly identified function of the DNA repair regulator CtIP, which promotes the stabilization of stalled replication forks through a mechanism involving p38α phosphorylation and subsequent PIN1‐mediated cis‐to‐trans isomerization of CtIP [38]. Of note, p38α inhibition sometimes induces cancer cells to enter senescence, sensitizing them to treatment with senolytic compounds [8].

Another interesting link has been established with the process so‐called anastasis, by which cancer cells may survive treatments with apoptotic drugs, such as TRAIL or paclitaxel. Anastatic cancer cells can secrete factors that promote drug resistance, tumor recurrence, and metastasis, a process that appears to be associated with sustained p38α activation even after the apoptotic stimulus has been removed [39].

The ability of p38α in several tumor‐associated cell types to control tumor immunity suggests that p38α inhibition may potentially affect the response to immunotherapies, such as adoptive T‐cell therapies [26] and checkpoint inhibitors. Notably, the combination of a p38α inhibitor with an antibody that activates OX40 (CD134), a costimulatory receptor in T cells, synergistically reduced metastatic growth and increased overall survival in mouse models of metastatic breast cancer. The combination therapy enhanced T‐cell activation, especially cytotoxic CD8^+^ T cells, and induced a robust, long‐lasting immunological memory [27]. Similarly, combining a p38α inhibitor with a PD‐L1 antibody has been shown to extend the survival of mice with temozolomide‐resistant glioblastoma [40].

## 5 Therapeutic targeting of p38α in cancer

The use of genetic and chemical approaches has evidenced pro‐tumorigenic roles of the p38α pathway in several cellular and animal models of cancer suggesting that targeting p38α signaling could be of clinical interest. Evidence from bioinformatic and immunohistochemical studies further support the therapeutic potential of p38α inhibitors, as the p38α pathway appears frequently upregulated in diverse types of human tumors, where higher activity is generally associated with reduced patient survival [9, 10, 32, 41, 42, 43, 44]. Of note, the significance of p38α in certain tumor contexts is not necessarily associated with higher activity compared to nonmalignant cells. Nevertheless, it is tempting to speculate that cancer cells with elevated p38α signaling might be more sensitive to the treatments involving p38α inhibitors. It is also increasingly evident that a deeper understanding of the tumor stroma will be crucial for developing more effective anticancer therapies. In this regard, a specific gene signature identified in mouse macrophages following p38α inhibition, characterized by the upregulation of IFNγ‐responsive factors, has been associated with improved overall survival in patients with certain types of breast cancer [27].

There are some cases in which monotherapy with a p38α inhibitor can impair cancer cell proliferation and reduce tumor growth effectively [8], but combination treatments usually show a much‐improved anticancer effect, suggesting that p38α inhibition may work best as an agent that sensitizes cancer cells to existing therapy regimens. This has been well‐documented in the case of chemotherapy drugs that induce DNA damage, but it is also emerging as a valuable possibility for specific therapies that either rewire signaling in the cancer cells or facilitate the targeting of cancer cells by immune cells.

A number of p38α chemical inhibitors have been developed that have good pharmacokinetic properties and remarkable potency, being able to strongly reduce kinase activity at low nanomolar concentrations, but have shown limited efficacy in clinical trials. In some cases, this is probably related to the development of toxicity in patients, which made it necessary to lower the doses of compound administered, thus reducing efficacy. It is also possible that we still do not understand how p38α functions in the cellular milieu. In particular, p38α interaction with other proteins and intracellular molecules may provide a quite different environment from the conditions normally used to test the inhibitors in kinase assays or in cellular systems with the pathway hyperactivated. Likewise, it is not understood how alternative signaling pathways, including other members of the p38 MAPK subfamily, may compensate for the blockade of p38α in the regulation of cancer cell viability. It should be noted that it is mostly unclear whether the toxicity observed in clinical trials with p38α inhibitors is due to off‐target effects or to nondisease‐related roles of p38α in organismal homeostasis.

The possibility that p38α inhibitors may have off‐target effects is an important consideration, and several publications have shown that p38α inhibitors widely used for research can target other proteins [45]. Recently, the p38α inhibitor ralimetinib (LY2228820), which advanced to phase II clinical trials in oncology, was shown to inhibit epidermal growth factor receptor (EGFR) kinase activity *in vitro*, albeit with up to 50‐fold lower potency than p38α. Structural studies support that ralimetinib can function as an ATP‐competitive inhibitor of EGFR, and treatment with ralimetinib impairs the proliferation of cancer cell lines driven by EGFR‐activating mutations [46]. While this work shows that ralimetinib can inhibit EGFR *in vitro* and in cells, it does not demonstrate that the anticancer effects of ralimetinib generally depend on EGFR inhibition rather than on p38α inhibition, except when cancer cell proliferation is driven by EGFR.

It is not surprising that in cancer cells whose viability does not depend on p38α, the response to p38α inhibitors is unaffected upon p38α downregulation. The argument that an inhibitor is not specific because it reduces the proliferation of cells that do not express the expected target is not necessarily correct. Cancer cells that are dependent on p38α should die upon p38α inactivation; however, some p38α KO cells still manage to proliferate, most likely because they have rewired their signaling networks so their viability is now independent of p38α. It should be noted that p38α inhibitors usually impair the proliferation of p38α KO cells at very high concentrations, sometimes > 100‐times the doses required to inhibit p38α activity, suggesting that those effects are indeed nonspecific. These observations do not preclude that the viability of certain cancer cells depends on p38α. In fact, to our knowledge, there is currently no conclusive evidence that the anticancer effects of p38α inhibitors involve targets other than p38α. Moreover, in several studies, the outcomes of chemical inhibitors have been corroborated through genetic inactivation of p38α.

Certainly, chemical compounds are unlikely to be 100% specific and it is very useful to identify proteins other than p38α whose activity can be modulated by p38α inhibitors, as this information will undoubtedly help to optimize the use of pharmacological p38α inhibitors to treat cancer and other diseases. It is also important to keep in mind that p38α inhibition or downregulation may result in different effects. For example, suppression of p38α signaling often induces upregulation of MKK6, a kinase capable of activating all p38 MAPKs but that binds to p38α with higher efficiency [5, 47], thereby potentially leading to different cellular outcomes depending on whether p38α is inhibited or absent.

An exciting recent development has been the generation of a new class of p38α inhibitors named ULTR‐p38i [8]. These compounds are predicted to have a longer target residence time, thereby ensuring a more robust inhibition of the p38α kinase activity. In fact, ULTR‐p38i monotherapy shows strong therapeutic effects in patient‐derived organoids and syngeneic mouse models of colorectal cancer, while previously developed ATP‐competitive inhibitors of p38α have minor effects in the same models. Interestingly, ULTR‐p38i strongly reduced colorectal cancer metastasis in mouse models, while showing no measurable toxicity in mice [8]. It is tempting to speculate that the long‐target‐residence‐time of these inhibitors would allow the kinase to stay inactive in a more continuous manner, avoiding the ON and OFF cycles of activity that may suffice for p38α to perform some cellular functions, especially when key substrates are dephosphorylated at a slower rate.

## 6 Conclusions

p38α plays a multifaceted role in fine‐tuning cellular processes and can be viewed as a general fail‐safe mechanism that helps maintain cell homeostasis. Although capable of regulating diverse functions, p38α's specific roles are highly context‐dependent, making its functions difficult to predict in individual cell types. This functional plasticity likely explains why p38α is co‐opted by certain cancer cells to support survival and proliferation through multiple mechanisms, while remaining rarely mutated in tumors despite exhibiting tumor‐suppressive functions in non‐malignant cells. Importantly, regulation of cancer cell viability is not a universal function of p38α, and there is evidence that proliferation of established cancer cell lines grown as monolayers is largely independent of p38α signaling.

Small‐molecule p38α inhibitors were originally developed for chronic inflammatory diseases, which require long‐term treatment. In contrast, cancer may represent a more suitable setting for their use, as p38α inhibitors could be applied acutely and in combination with other therapies. Despite promising preclinical results, however, at least four p38α inhibitors have entered clinical trials for solid and hematological malignancies, with none progressing beyond phase II [7] (https://www.clinicaltrials.gov/study/NCT04074967).

These outcomes suggest that patient stratification and improved preclinical models are needed to better identify tumors that may benefit from p38α targeting. In parallel, alternative strategies should be explored, including compounds that mimic the antiproliferative effects of p38α phosphorylation on specific substrates, promote p38α degradation, or enable cell type–specific targeting to minimize systemic toxicity (reviewed by [5]). Inhibitors with longer residence times also warrant further investigation.

Because p38α inhibitors likely affect multiple cell types within the tumor microenvironment, a deeper understanding of p38α‐mediated communication between cancer and stromal cells is essential. Defining the cellular and genetic contexts that dictate p38α dependency, as well as compensatory signaling pathways, should guide the development of more selective therapies and effective combination strategies to fully exploit the therapeutic potential of targeting p38α in cancer.

## Conflict of interest

The author declares no conflict of interest.

## Author contributions

ARN conceived and wrote the paper.
